# Metal Oxide/TiO_2_ Hybrid Nanotubes Fabricated through the Organogel Route

**DOI:** 10.3390/gels3030024

**Published:** 2017-06-22

**Authors:** Masahiro Suzuki, Keita Tanaka, Yukie Kato, Kenji Hanabusa

**Affiliations:** Graduate School of Science and Technology, Shinshu University, Ueda, Nagano 386-8567, Japan; 14f3072k@shinshu-u.ac.jp (K.T.); 14f3015a@shinshu-u.ac.jp (Y.K.); hanaken@shinshu-u.ac.jp (K.H.)

**Keywords:** low-molecular-weight gelator, titanium dioxide, nanofiber, nanotube, supramolecular chemistry, sol-gel polymerization, template-synthesis

## Abstract

Titanium dioxide (TiO_2_) nanotube and its hybrid nanotubes (with various metal oxides such as Ta_2_O_5_, Nb_2_O_5_, ZrO_2,_ and SiO_2_) were fabricated by the sol-gel polymerization in the ethanol gels formed by simple l-lysine-based organogelator. The self-assembled nanofibers (gel fibers) formed by the gelator functioned as a template. The different calcination temperatures gave TiO_2_ nanotubes with various crystalline structures; e.g., anatase TiO_2_ nanotube was obtained by calcination at 600 °C, and rutile TiO_2_ nanotube was fabricated at a calcination temperature of 750 °C. In the metal oxide/TiO_2_ hybrid nanotubes, the metal oxide species were uniformly dispersed in the TiO_2_ nanotube, and the percent content of metal oxide species was found to correspond closely to the feed ratio of the raw materials. This result indicated that the composition ratio of hybrid nanotubes was controllable by the feed ratio of the raw materials. It was found that the metal oxide species inhibited the crystalline phase transition of TiO_2_ from anatase to rutile. Furthermore, the success of the hybridization of other metal oxides (except for TiO_2_) indicated the usefulness of the organogel route as one of the fabrication methods of metal oxide nanotubes.

## 1. Introduction

Low-molecular-weight gelators and their supramolecular gels have been actively investigated, and many gelators have been reported [1,2,3,4,5,6,7,8,9,10,11]. In the supramolecular gels, most of low-molecular-weight gelators create three-dimensional networks by the entangling of self-assembled nanofibers through non-covalent interactions [1,2,3,4,5,6,7,8,9,10,11]. Besides basic studies (such as gelation property, gel morphology, and the physical property of gels), extensive research related to the application of the gels has been investigated: for example, functional and stimuli responsive gels [12,13,14], photonic and electronic devices [15,16,17,18], biomaterials [19,20,21,22], and others [23,24,25,26,27]. The nanostructures of self-assembled nanofibers created in the supramolecular gels have a high utilization value as a template because the gelators can simply construct various nanostructures such as twisted nanoribbons, coiled nanorods, and helical nanofibers and nanotubes [10]. Nanostructured metal oxides of titanium, tantalum, vanadium, zirconium, and niobium are generally accepted as the new materials in electronics and catalysts [28,29]. Shinkai and co-workers are a pioneer in the template-synthesis of metal oxides using low-molecular-weight gelators [30,31]. The nanotubes and nanofibers of SiO_2_, TiO_2_, ZrO_2_, Ta_2_O_5_, Nb_2_O_5_, and vanadium oxide have been successfully fabricated using gelators [11,32,33,34,35,36,37,38,39,40].

TiO_2_ is one of the best semiconducting photocatalysts because it has good photoreactivity, nontoxicity, stability, and low cost [28,29,41,42]. To improve the photocatalytic activity of TiO_2_, some methods have been suggested, e.g., a fabrication of nanostructured TiO_2_ and hybridization with other metal oxides [42]. There are many reports on the synthesis of nanostructured TiO_2_ [43,44,45] and hybridization with platinum, copper, tungsten oxide, etc. [46,47,48,49]. It is well-known that the TiO_2_ nanotubes are frequently fabricated by hydro/solvothermal methods, anodization, templates, and electrospinning [50]. For example, TiO_2_ arrays were fabricated using anodic aluminum oxide or zinc oxide nanowire as templates [51]. As mentioned above, the fabrication of metal oxides using organogelators has been reported, while the template-synthesis of hybrid metal oxides have hardly been reported. To obtain the metal oxides at low cost, it is necessary to minimize the cost of the disposable gelators. We have reported the fabrication of TiO_2_ nanotube using the l-lysine organogelator, which can be simply and inexpensively prepared [35,52]. In this paper, we describe the fabrication and characterization of TiO_2_ nanotubes through the organogel route, using the simply synthesized l-lysine organogelator (Gelator 1) and the hybridization with metal oxides into TiO_2_ nanotubes.

## 2. Results and Discussion

### 2.1. Preparation of Gelator 1

**Scheme 1:**
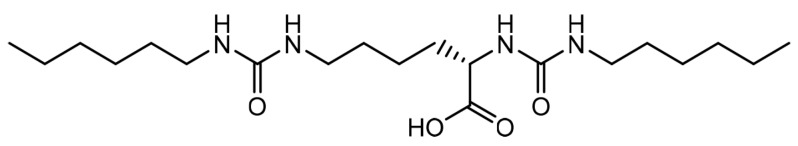
Chemical structure of Gelator 1.

Gelator 1 was a very simple and powerful gelator and simply synthesized in high yields [35,52]; Methyl 2,6-diisocyanatohexanoate (l-lysine diisocyanate methyl ester) was used as a starting material and reacted with hexylamine. Gelator 1 was obtained by the deesterification of the methyl ester in NaOH solution and then acidification by HCl (93% yield) (Scheme 1). Gelator 1 functioned as a good organogelator that formed the organogels in many organic solvents and oils.

### 2.2. Fabrication of TiO_2_ Nanotubes

The minimum gelation concentration (MGC) of gelator 1 was 30 mg/mL in ethanol. The MGC value for a mixture of Ti(O^i^Pr)_4_ and ethanol (1:1 *v*/*v*) was 50 mg/mL. Considering the effect of the addition of propylamine, it was decided that the concentration of gelator 1 was 61 mg (0.15 mmol). The preliminary experiments demonstrated that the best experimental condition for the fabrication of metal oxides nanotubes; the molar ratio of the raw materials (such as gelator, metal alkoxides, and propylamine) was 0.15:0.51:0.30 (61 mg of gelator, 150 μL of Ti(O^i^Pr)_4_, and 25.1 μL of C_3_H_7_NH_2_) in 0.85 mL of solvent) [35]. The sol-gel polymerization in 1,4-dioxane gel gave a TiO_2_ nanowire (not nanotube). We have reported that the TiO_2_ nanowires were fabricated by using L-isoleucine gelator [39]. Although the l-isoleucine gelator formed the self-assembled nanofibers with a diameter of several tens of nanometers in the dioxane gel, the inner diameter of the TiO_2_ nanotubes obtained was 1–10 nanometers before calcination. However, the nanotubes changed into nanowires during calcination over 400 °C. This is attributed to the fact that nanotubes are shrunken by calcination. In the present case, the TiO_2_ nanowire was formed by the same process. Therefore, we used ethanol as a solvent.

Ten samples (in which the sol-gel polymerization was performed at various reaction times) were evaluated to find a suitable sol-gel polymerization time: 1 to 10 days. When the sol-gel polymerization was performed in the reaction times from 6 to 10 days, the yields of the nanotubes (based on Ti(O^i^Pr)_4_) were almost the same (92–95%). In contrast, in 1–5 days the yields were 10–70%. Therefore, the fabrication of TiO_2_ nanotubes was carried out in 7 days of sol-gel polymerization time. Under the experimental conditions, the nanotubes are produced in high yields. Sometimes, small fragments of broken tubes are observed in SEM (scanning electron microscope) and TEM (transmission electron microscope) measurements (Figure S1).

**Figure 1:**
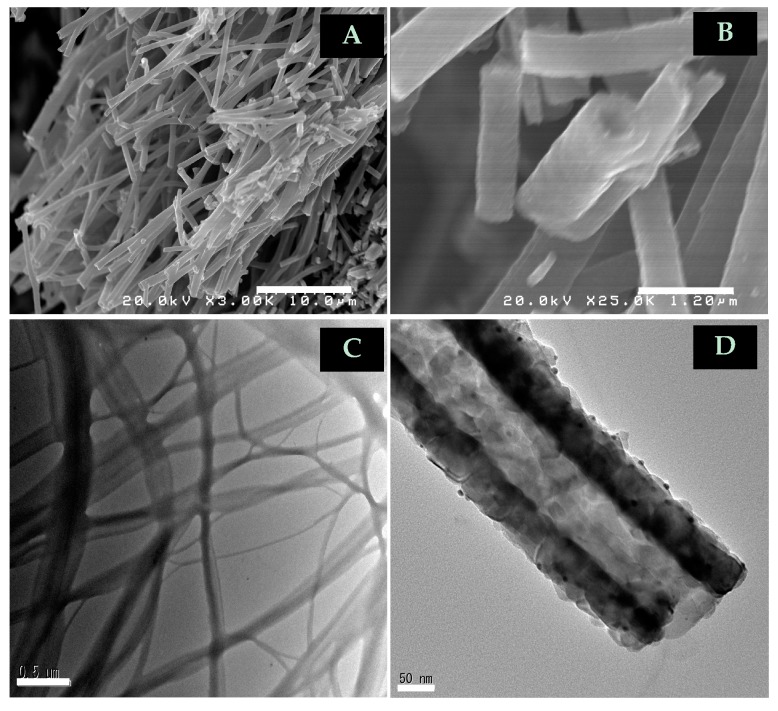
FE-SEM images of TiO_2_ nanotubes (**A**,**B**) and TEM (transmission electron microscope ) images of gel fibers, prepared from ethanol gel of gelator 1 (**C**) and TiO_2_ nanotubes (**D**). Scale bars are 10 μm (**A**); 1.2 μm (**B**); 0.5 μm (**C**); and 50 nm (**D**). The TiO_2_ nanotubes were calcined at 600 °C.

Figure 1 shows the field emission scanning electron microscope (FE-SEM) images of TiO_2_ nanotube (A,B), and TEM images of dry sample prepared from the ethanol gel of gelator 1 (C) and TiO_2_ nanotube (D) where the nanotubes were calcined at 600 °C. Gelator 1 created the three-dimensional networks entangling the self-assembled nanofibers with the diameter of 10–300 nm (image C). The TiO_2_ nanotubes obtained by the sol-gel polymerization in the ethanol gel had a diameter of 400 nm to 1 μm, and a length of a hundred to several tens of micrometers; in particular, the inner diameter was 100–600 nm. These results indicate that the nanofibers function as a template.

### 2.3. Effect of Calcination Temperatures on TiO_2_ Nanotubes

**Figure 2:**
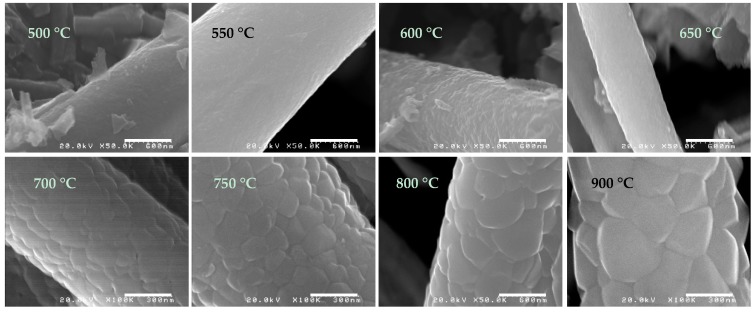
FE-SEM images of surfaces of TiO_2_ nanotubes, fabricated at various calcination temperatures (500–900 °C). Scale bars are 600 nm for 500–650 and 800 °C, and 300 nm for 700, 750 and 900 °C.

The structures of TiO_2_ nanotubes were affected by the calcination temperatures. When the calcination temperature was below 750 °C, the TiO_2_ nanotube maintained its nanotube structure. The nanotube was partly sintered and decomposed at the calcination temperature of 800 °C. The calcination temperature of 900 °C promoted the sintering and collapse of nanotubes. In addition, the surface morphology of the TiO_2_ nanotubes dramatically changed with the increasing calcination temperature. Figure 2 shows the FE-SEM images of surfaces of TiO_2_ nanotubes fabricated at various calcination temperatures. The surfaces of nanotubes had a smooth structure and hardly changed up to 650 °C of calcination temperature. With increasing calcination temperatures (over 650 °C), the surfaces with microstructures gradually changed into a large mass structure (more than 800 °C). As such, the change of the surface structure is obviously caused by sintering.

**Figure 3:**
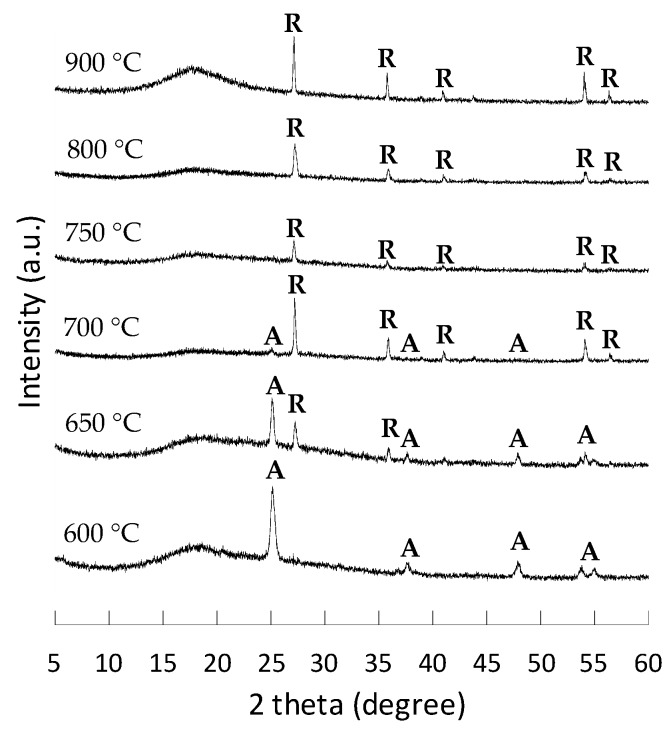
Powder XRD patterns of TiO_2_ nanotubes fabricated at various calcination temperatures.

It is well-known that a TiO_2_ changes its crystalline structure in calcination temperatures [29,39]. The change in the crystalline structure of the TiO_2_ nanotubes during calcination was evaluated by X-ray diffraction (XRD) analysis. Figure 3 shows the XRD patterns of TiO_2_ nanotubes, fabricated at various calcination temperatures. The crystalline structure of the nanotubes changed from anatase to rutile with the increasing calcination temperature, and the change occurred at 650–700 °C. The nanotubes calcined at 650 and 700 °C had the crystalline structures of a mixture of anatase and rutile (anatase:rutile = 4:6 at 650 °C and = 1:9 at 700 °C). The anatase nanotube was obtained by calcination at 600 °C, and the rutile nanotube was fabricated by a calcination temperature of 750 °C.

Furthermore, the Brunauer-Emmett-Teller (BET) surface areas were listed in Table 1. The BET surface areas depended upon the calcination temperature (in other words, the crystalline structure and nanotube structure); the surface areas decreased with the increasing calcination temperature. When the crystalline structure changed from anatase to rutile (maintaining their nanotube structures), the BET surface area decreased. At high calcination temperatures (more than 800 °C), the nanotube was decomposed by sintering, which lead to the dramatic decrease in the BET surface area.

### 2.4. Fabrication of Other Metal Oxides

**Figure 4:**
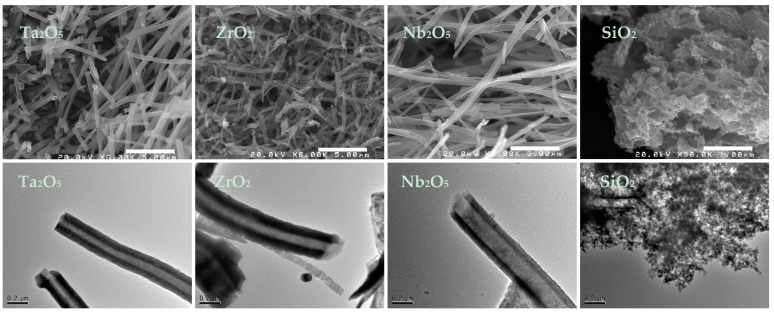
FE-SEM (**upper**) and TEM (**lower**) images of Ta_2_O_5_, ZrO_2_, Nb_2_O_5_, and SiO_2_ fabricated in ethanol gels. Scale bars are 5 μm for Ta_2_O_5_, ZrO_2_ and Nb_2_O_5_; 5 μm for SiO_2_ in SEM; and 0.2 μm in TEM images.

The fabrication of tantalum(V) oxide (Ta_2_O_5_), zirconium(IV) oxide (ZrO_2_), niobium(V) oxide (Nb_2_O_5_), and silica (SiO_2_), was carried out under the same experimental conditions as the TiO_2_ nanotube. In the present cases, we used tantalum pentaethoxide Ta(OEt)_5_, zirconium tetrabutoxide Zr(OBu)_4_, niobium pentaethoxide Nb(OEt)_5_, and tetraethoxysilane Si(OEt)_4_ as starting materials. As a control experiment, the sol-gel polymerization without gelator 1 was performed under the same conditions. The nanoparticles of Ta_2_O_5_, ZrO_2_, and Nb_2_O_5_ were fabricated, while the non-nanostructured SiO_2_ was obtained (Figure S2); namely, the nanotubes were not fabricated without gelator 1. Figure 4 shows the FE-SEM images of Ta_2_O_5_, ZrO_2_, Nb_2_O_5_, and SiO_2_ fabricated in the ethanol gel. The nanotubes of Ta_2_O_5_, ZrO_2_, and Nb_2_O_5_ were obviously obtained, and they had a diameter of 100–500 nm and a length of several tens of micrometers, which were almost the same as the TiO_2_ nanotube. Therefore, it is indicated that the gel fibers function as a good template for Ta_2_O_5_, ZrO_2_, and Nb_2_O_5_. In contrast, a small amount of SiO_2_ nanotube was obtained by the sol-gel polymerization in the organogels, but most of SiO_2_ had the form of small blocks similar to that obtained without gelator. The gel fibers could not function as the effective template for the sol-gel polymerization of tetraethoxysilane (TEOS) under the experimental conditions. This is probably that reason that the rate of sol-gel polymerization of TEOS is fast. The nanotubes of SiO_2_ can be obtained by the control of the sol-gel polymerization rate. In addition, the XRD patterns of these obtained nanotubes were the same as the typical metal oxides (Figure S3).

### 2.5. Fabrication of Ta_2_O_5_/TiO_2_ Hybrid Nanotubes

**Figure 5:**
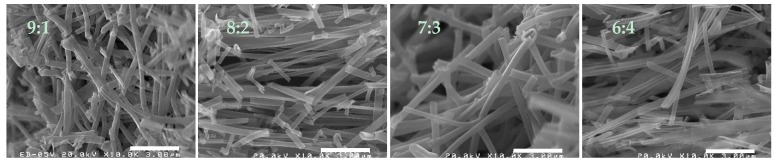
FE-SEM images of Ta_2_O_5_/TiO_2_ hybrid nanotubes fabricated in ethanol gels. The ratios of Ti and Ta are 9:1, 8:2, 7:3, and 6:4 from left. Calcination temperature is 600 °C. Scale bars are 3 μm.

The Ta_2_O_5_/TiO_2_ hybrid nanotube was fabricated using the organogel route. Figure 5 shows the FE-SEM images of samples fabricated by the sol-gel polymerization in the ethanol gels, containing various feed ratios of Ti(O^i^Pr)_4_ and Ta(OEt)_5_ (9:1 to 6:4). In all ratios, the nanotubes were obtained, which were almost the same sizes as the TiO_2_ nanotube. This result indicates that the gel fibers also function as the template for the sol-gel polymerization of the mixture of Ti(O^i^Pr)_4_ and Ta(OEt)_5_. The Ta_2_O_5_/TiO_2_ hybrid nanotubes were characterized by an energy dispersive x-ray (EDX) analysis. The peaks, arising from Ti and Ta, were observed in the EDX spectra for all samples, and the element mapping analysis demonstrated that the Ta_2_O_5_ species were uniformly dispersed in the TiO_2_ nanotubes (Figures S4 and S5). In addition, the percent content of Ta_2_O_5_ species in the Ta_2_O_5_/TiO_2_ hybrid nanotube was found to correspond closely to the feed ratio of the metallic precursors as raw materials. This enables the fabrication of hybrid nanotubes of a different composition ratio when selecting the feed ratio of the raw materials.

Interestingly, the hybridization with Ta_2_O_5_ affected the crystalline structure of the TiO_2_ nanotubes. As mentioned above, the TiO_2_ nanotubes changed their crystalline structures from anatase into rutile by calcination at 650–700 °C. In contrast, the Ta_2_O_5_/TiO_2_ hybrid nanotubes had the anatase crystalline structure even when calcining at 900 °C. The fact clearly indicates the inhibition of the crystalline phase transition of TiO_2_ by Ta_2_O_5_ species, and proves the uniform dispersion of Ta_2_O_5_ species in the TiO_2_ (Figure S6).

### 2.6. Hybridization of Other Metal Oxides into TiO_2_

**Figure 6:**
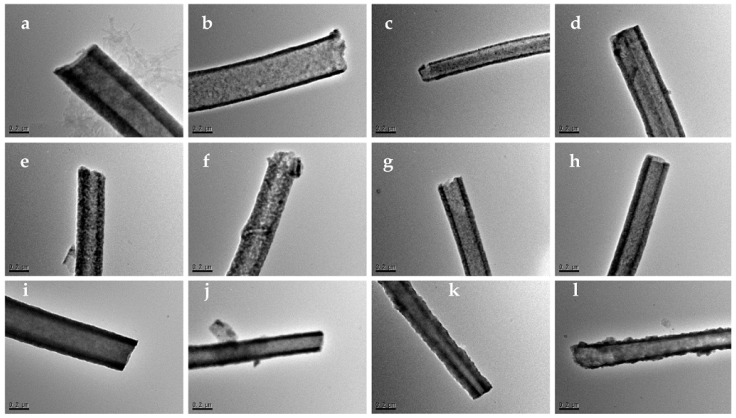
TEM images of metal oxide/TiO_2_ hybrid nanotubes (**a**–**d**: Zr, **e**–**h**: Nb, and **i**–**l**: Si). Ti:Zr = 9:1 (**a**); 8:2 (**b**); 7:3 (**c**) and 6:4 (**d**); Ti:Nb = 9:1 (**e**), 8:2 (**f**), 7:3 (**g**) and 6:4 (**h**); Ti:Si = 9:1 (**i**), 8:2 (**j**), 7:3 (**k**) and 6:4 (**l**). Scale bars are 0.2 μm.

The TiO_2_ nanotubes hybridized with other metal oxides (ZrO_2_, Nb_2_O_5_, and SiO_2_) were fabricated in the ethanol gels. Figure 6 shows the TEM images of samples fabricated in the ethanol gel containing two metallic precursors. For all samples, the nanotube structures were observed. The XPS analysis proved that the structures of metals in the TiO_2_ nanotubes were their oxides (ZrO_2_, Nb_2_O_5_, and SiO_2_, Figure S7). The EDX and element mapping analyses demonstrated that the metal oxides hybridized were uniformly dispersed into the TiO_2_ nanotubes (Figures S8–S10). The XRD profiles for the ZrO_2_/TiO_2_ hybrid nanotubes showed that the crystalline structure of the hybrid nanotubes tended to become amorphous with increasing content of ZrO_2_. With increasing ZrO_2_ contents, the respective XRD peaks arising from ZrO_2_ and TiO_2_ disappeared, and a new broad peak appeared around 2θ = 20° (Figure S7). For the Nb_2_O_5_/TiO_2_ hybrid nanotubes, the peaks of Nb_2_O_5_ (in addition to a broad XRD peak) appeared, but the peak of TiO_2_ disappeared. These facts prove that the ZrO_2_ and Nb_2_O_5_ are uniformly hybridized with TiO_2_.

Surprisingly, the SiO_2_/TiO_2_ hybrid nanotubes were obtained by the organogel route (up to 6:4 = Ti:Si), although the SiO_2_ nanotubes were hardly fabricated. In addition, the silica was uniformly dispersed into the TiO_2_ nanotube (Figure S10). The fabrication of the SiO_2_/TiO_2_ hybrid nanotubes was achieved by the sol-gel copolymerization. This is attributed to the fact that the concentration of the SiO_2_ precursor is relatively low and is easy to react with the TiO_2_ precursor. In the ratio of 5:5, however, the yield of the nanotube significantly decreased. The sample obtained was a mixture of nanotubes and large masses and the SEM image was similar to that of silica shown in Figure 4. Therefore, it was found that the gel fibers could not function as the template in the high TEOS ratios (more than 5:5).

### 2.7. Hybridization of Metal Oxides Except for TiO_2_

**Figure 7:**
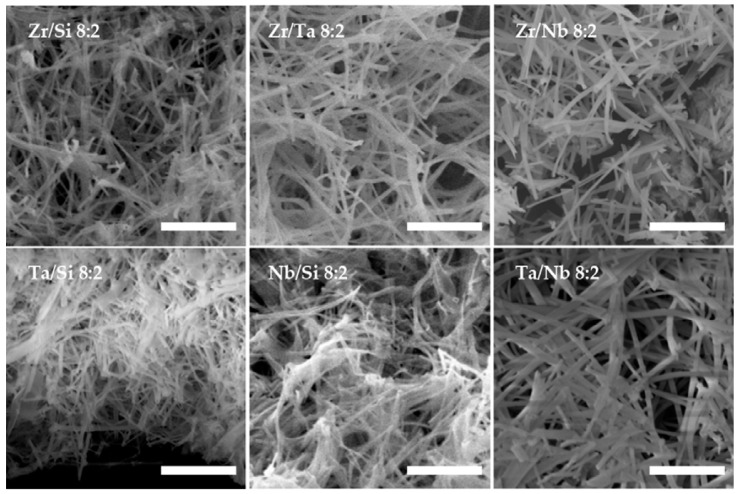
FE-SEM images of hybrid nanotubes of ZrO_2_/SiO_2_ (8:2), ZrO_2_/Ta_2_O_5_ (8:2), ZrO_2_/Nb_2_O_5_ (8:2), Ta_2_O_5_/SiO_2_ (8:2), Nb_2_O_5_/SiO_2_ (8:2), and Ta_2_O_5_/Nb_2_O_5_ (8:2). Scale bars are 5 μm.

The hybridizations between other metal oxides (except for TiO_2_) were also successful; the sol-gel copolymerization in the ethanol gel gave the hybrid nanotubes of SiO_2_/ZrO_2_, Ta_2_O_5_/ZrO_2_, Nb_2_O_5_/ZrO_2_, SiO_2_/Ta_2_O_5_, Nb_2_O_5_/Ta_2_O_5_, and SiO_2_/Nb_2_O_5_ (as shown in Figure 7). These hybrid nanotubes were several tens of micrometers in length, with an outer diameter of several hundreds of nanometers, and an inner diameter of 100–600 nm. These values are almost the same as each metal oxide (except for SiO_2)_. The hybridizations with SiO_2_ were achieved when the ratios of metal and Si were 8:2 or less (Figures S11 and S12).

## 3. Conclusions

In conclusion, we revealed that the fabrication of TiO_2_ nanotubes and hybridization with other metal oxides can be accomplished by the sol-gel method using a simple l-lysine organogelator as a template. The self-assembled nanofibers formed by l-lysine organogelator functioned as a template, and metal oxide nanotubes several tens of micrometers in length, an outer diameter of several hundreds of nanometers, and an inner diameter of 100–500 nm were fabricated. The crystalline structures of pure TiO_2_ nanotubes changed from anatase into rutile with increasing calcination temperatures. The hybrid nanotubes of TiO_2_ with Ta_2_O_5_, ZrO_2_, Nb_2_O_5_, and SiO_2_ were fabricated by the sol-gel polymerization in ethanol gels containing two raw materials. The hybridized Ta_2_O_5_, ZrO_2_, Nb_2_O_5_, and SiO_2_ were uniformly dispersed into the TiO_2_ nanotube, and their percentage contents closely corresponded to the feed ratio of raw materials. These results indicated that the TiO_2_ hybrid nanotubes, with various contents of metal oxides, were easily fabricated by only changing the feed contents. The property of TiO_2_ nanotubes was changed by the hybridization of metal oxides; e.g., the surface areas increased, and the crystalline phase transition temperature from anatase to rutile became high. Furthermore, the hybridization of metal oxides (except for TiO_2_) was successful using the organogel route.

## 4. Materials and Methods

### 4.1. Materials

Gelator 1 was prepared according to the literature [35,52]. The other chemicals were of the highest commercially available grade, and used without further purification: Titanium(IV) tetra(isopropoxide) (from Wako Pure Chemical Industries, Tokyo, Japan, 95%), tetraethyl orthosilicate (Wako, 95%), zirconium(IV) *n*-butoxide (Wako, 80 wt % in *n*-BuOH), niobium(V) ethoxide (Wako, 99.9%), tantalum(V) ethoxide (Sigma-Ardrich Japan, Tokyo, Japan, 99.9%), propylamine (Wako, 98%, S), and ethanol (Wako, 95%, S). All solvents used in the syntheses were purified, dried, or fleshly distilled as required.

### 4.2. Sol-Gel Polymerization

The sol-gel polymerization was performed using the modified method in previous reports [34,35,36]. The typical procedure was as follows: the metal alkoxide and propylamine were added to the ethanol gel in a test tube, and the mixture was heated at 80 °C until the clear solution was obtained; the resulting hot solution was then cooled and the gel was obtained. The gel was allowed to stand at 25 °C for a set of period of time (1 to 10 days). The resulting dried gels were washed with chloroform to remove the gelator and dried at 40 °C for 12 h. Finally, the dried samples were heated at 90 °C for 2 h and then over 500 °C for 3 h. The hybridization of metal oxides (Ta_2_O_5_, ZrO_2_, Nb_2_O_5_, and SiO_2_) into TiO_2_ was performed by using the organogel route.

### 4.3. Instrumentation and Techniques

The elemental analysis was performed using a Perkin-Elmer series II CHNS/O analyzer 2400 (from Parkin-Elmer Japan Co., Ltd., Tokyo, Japan). The FT-IR spectra were recorded on a JASCO FS-420 spectrometer (JASCO, Tokyo, Japan). The ^1^H NMR spectra were measured using a Bruker AVANCE 400 spectrometer with TMS (from Bruker Biospin K.K., Yokohama, Japan). The transmission electron microscope (TEM) images were obtained by using a JEOL JEM-2010 electron microscope at 200 kV (from JEOL Ltd., Tokyo, Japan). The field emission scanning electron microscope (FE-SEM) observations were carried out using a Hitachi S-5000 field emission scanning electron microscope (from Hitachi High-Technologies Co., Tokyo, Japan). Energy dispersed X-ray spectroscopy (EDX, HORIBA EX200) was used to obtain elemental analysis and element mapping (from Horiba Ltd., Kyoto, Japan). X-ray photoelectron spectroscope (XPS) characterization was performed using Mg Κα radiation at 15 kV and 10 mA on a KARATOS AZIS-ULTRA DLD (Karatos Analytical Ltd., Shimadzu Corporation, Kyoto, Japan). Products were characterized by powder X-ray diffraction, using Cu Κα radiation (λ = 1.5418 Å) at 40 kV and 150 mA in a range of 3°–60° on a Rigaku wide-angle X-ray diffractometer type Rad-rX (from Rigaku Co., Tokyo, Japan). The Brunauer-Emmett-Teller (BET) surface area was measured using nitrogen adsorption on a Shimadzu Gemini2375 (from Shimadzu Co., Kyoto, Japan).

## Figures and Tables

**Table 1:** Properties of TiO_2_ nanotubes fabricated at various calcination temperatures.

Calcination	Nanostructure	Crystal	BET Surface Area
550 °C	Nanotube	Anatase	19 m^2^/g
600 °C	Nanotube	Anatase	18 m^2^/g
650 °C	Nanotube	Anatase/Rutile	13 m^2^/g
700 °C	Nanotube	Anatase/Rutile	12 m^2^/g
750 °C	Nanotube	Rutile	10 m^2^/g
800 °C	Partly collapse	Rutile	5 m^2^/g
900 °C	Collapse	Rutile	4 m^2^/g

## Supplementary Materials

The following are available online at http://www.mdpi.com/2310-2861/3/3/24/s1, Synthesis of Gelator 1: Figure S1: FE-SEM of TiO_2_ nanotubes and small fragments; Figure S2: SEM images of Ta_2_O_5_, ZrO_2_, Nb_2_O_5_, and SiO_2_; Figure S3: XRD patterns of Ta_2_O_5_, ZrO_2_, Nb_2_O_5_, and SiO_2_ nanotubes; Figure S4: EDX spectra and element mappings of Ta_2_O_5_/TiO_2_ hybrid nanotubes; Figure S5: EDX spectra and element mappings of Ta_2_O_5_/TiO_2_ hybrid nanotubes; Figure S6: XRD patterns of Ta_2_O_5_/TiO_2_ hybrid nanotubes; Figure S7: XRD patterns of ZrO_2_/TiO_2_, Nb_2_O_5_/TiO_2_, and SiO_2_/TiO_2_ hybrid nanotubes; Figure S8: EDX spectra and element mappings of ZrO_2_/TiO_2_ hybrid nanotubes; Figure S9: EDX spectra and element mappings of Nb_2_O_5_/TiO_2_ hybrid nanotubes; Figure S10: EDX spectra and element mappings of SiO_2_/TiO_2_ hybrid nanotubes; Figure S11: EDX spectra and element mappings of ZrO_2_/SiO_2_ hybrid nanotubes; Figure S12: EDX spectra and element mappings of ZrO_2_/Ta_2_O_5_ hybrid nanotubes.
