# Supplementary material for: Metal Oxide/TiO2 Hybrid Nanotubes Fabricated through the Organogel Route
Source: Gels. 2017 Jun 22;3(3):24. doi: 10.3390/gels3030024 (PMC6318635; doi:10.3390/gels3030024)
Supplement: Supplementary file 1 [file gels-03-00024-s001.pdf]

Supplementary Materials

Metal Oxide/TiO_2 Hybrid Nanotubes Fabricated through the Organogel Route

Masahiro Suzuki, Keita Tanaka, Yukie Kato and Kenji Hanabusa

Contents

Synthesis of Gelator 1.

Figure S1. SEM images of TiO_2, nanotubes and small fragments of TiO_2.

Figure S2. SEM images of Ta_2O_5, ZrO_2, Nb_2O_5, and SiO_2.

Figure S3. XRD patterns of Ta_2O_5, ZrO_2, Nb_2O_5, and SiO_2 nanotubes

Figure S4. EDX spectra and element mappings of Ta_2O_5/TiO_2 hybrid nanotubes.

Figure S5. EDX spectra and element mappings of Ta_2O_5/TiO_2 hybrid nanotubes.

Figure S6. XRD patterns of Ta_2O_5/TiO_2 hybrid nanotubes.

Figure S7. XRD patterns of ZrO_2/TiO_2, Nb_2O_5/TiO_2, and SiO_2/TiO_2 hybrid nanotubes.

Figure S8. EDX spectra and element mappings of ZrO_2/TiO_2 hybrid nanotubes.

Figure S9. EDX spectra and element mappings of Nb_2O_5/TiO_2 hybrid nanotubes.

Figure S10. EDX spectra and element mappings of SiO_2/TiO_2 hybrid nanotubes.

Figure S11. EDX spectra and element mappings of ZrO_2/SiO_2 hybrid nanotube.

Figure S12. EDX spectra and element mappings of ZrO_2/Ta_2O_5 hybrid nanotube.

Synthesis of gelator 1

N^{ε}–Bis(hexylaminocarbonyl)–L–lysine methyl ester (10 mmol) was dissolved in MeOH–H_2O (400 mL–20 mL) and 4M NaOH (5 mL) was added. The reaction mixture was heated at 70°C for 24 h. The resulting solution was evaporated to ca. 100 mL, and water (600 mL) was added. The aqueous solution was adjusted to ca. pH = 1 by addition of 6M HCl with vigorous stirring. The white precipitate was filtered, washed with water, and then dried. The product was obtained by recrystallization from MeOH-ethyl acetate (92%). m.p. = 167-169 °C. IR (KBr): 3334 cm^{-1} (νN–H), 1782 cm^{-1}, 1701 cm^{-1} (νC=O, CO_2H), 1621 cm^{-1} (νC=O, urea), 1582 cm^{-1} (δN–H); ^{1}H-NMR (400 MHz, $CDCl_3$, TMS): δ = 0.87 (t, J = 6.6Hz, 6H), 1.27 (br, 14H), 1.38–1.51 (m, 6H), 1.71–1.81 (m, 2H), 3.11 (br, 6H), 4.26 (br, 1H), 5.41 (br, 1H), 5.64 (br, 1H), 5.89 (br, 1H), 6.19 (br, 1H). Elemental analysis calcd (%) for $C_{20}H_{40}N_4O_4$ (400.56): C, 59.97; H, 10.07; N, 13.99. Found: C, 60.66; H, 10.61; N, 14.13.

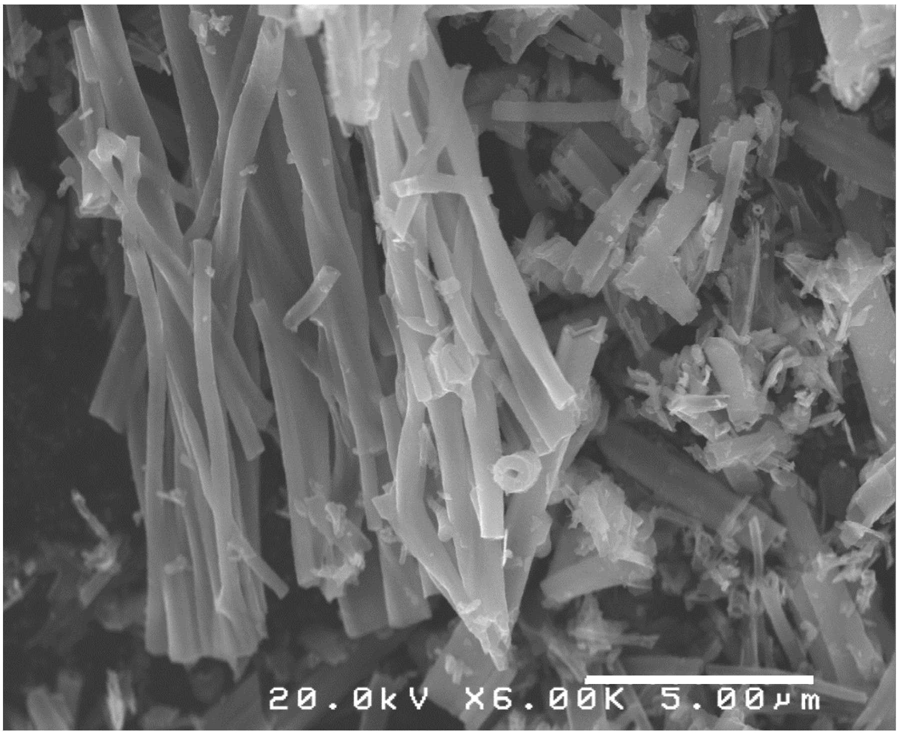

Figure S1. SEM image of TiO_2 nanotube and small fragments of TiO_2.

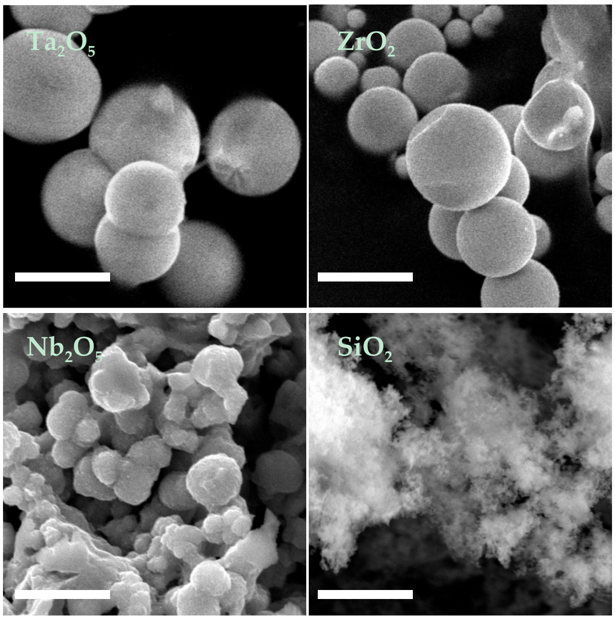

Figure S2. SEM images of Ta_2O_5, ZrO_2, Nb_2O_5, and SiO_2 fabricated without gelator. Scale bars are 5 μm.

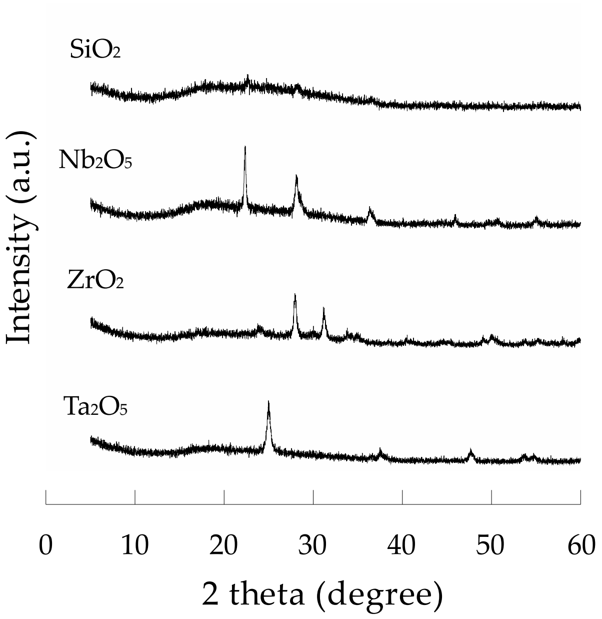

Figure S3. XRD patterns of Ta_2O_5, ZrO_2, Nb_2O_5, and SiO_2 nanotubes fabricated in ethanol gel.

Ti:Ta = 9:1

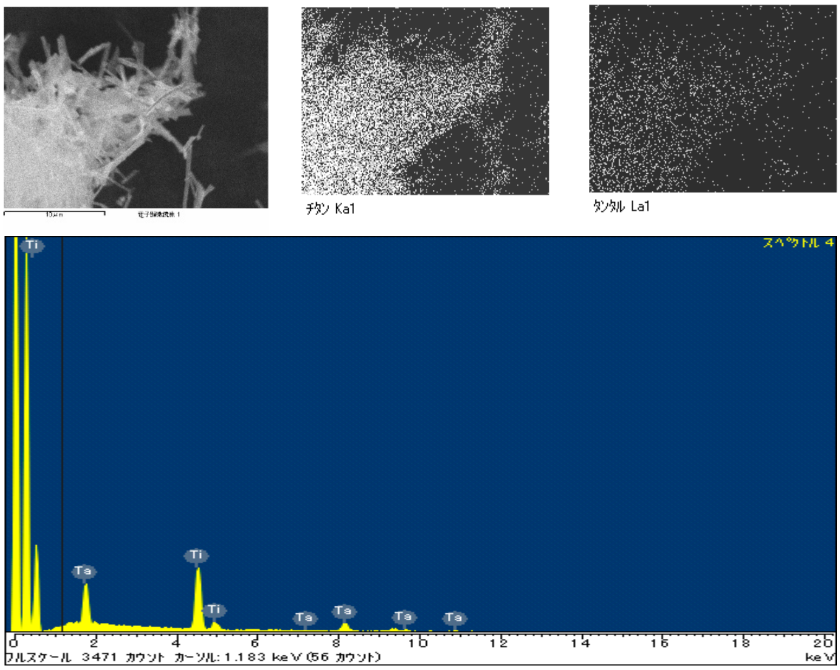

Ti:Ta = 8:2

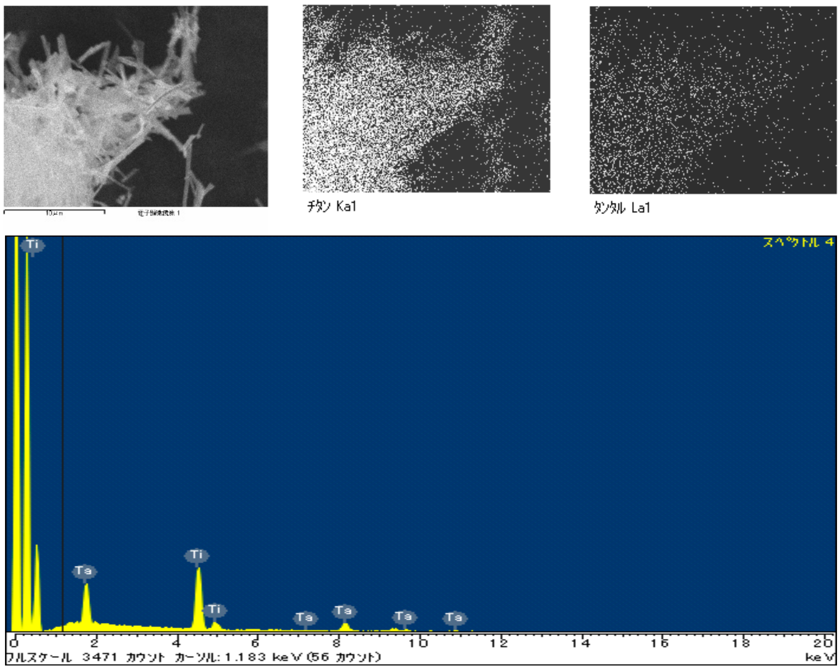

Figure S4. EDX spectra and element mappings of Ta_2O_5/TiO_2 hybrid nanotubes. Upper: Ti:Ta = 9:1; lower: Ti:Ta = 8:2. Scale bars are 10 μm.

Ti:Ta = 7:3

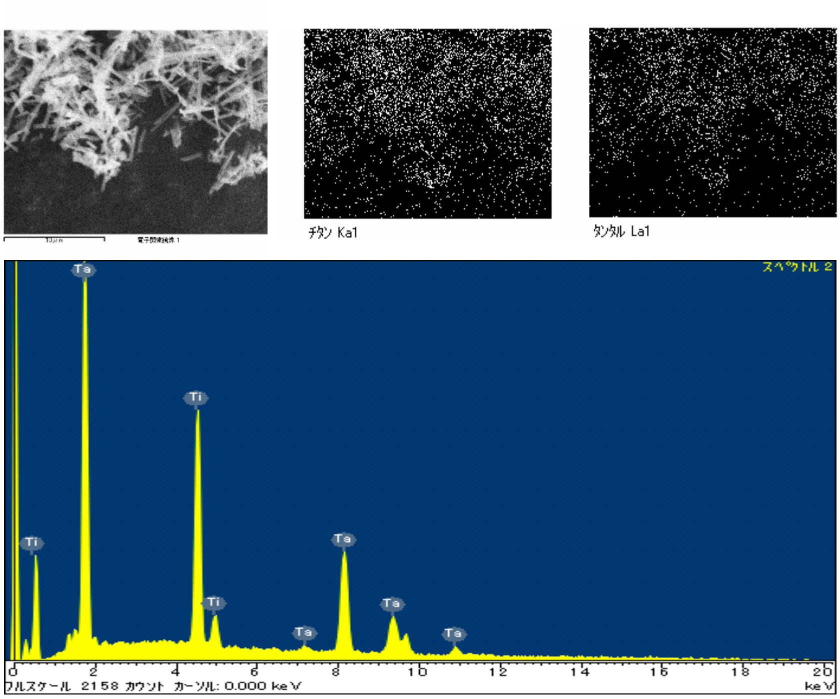

Ti:Ta = 6:4

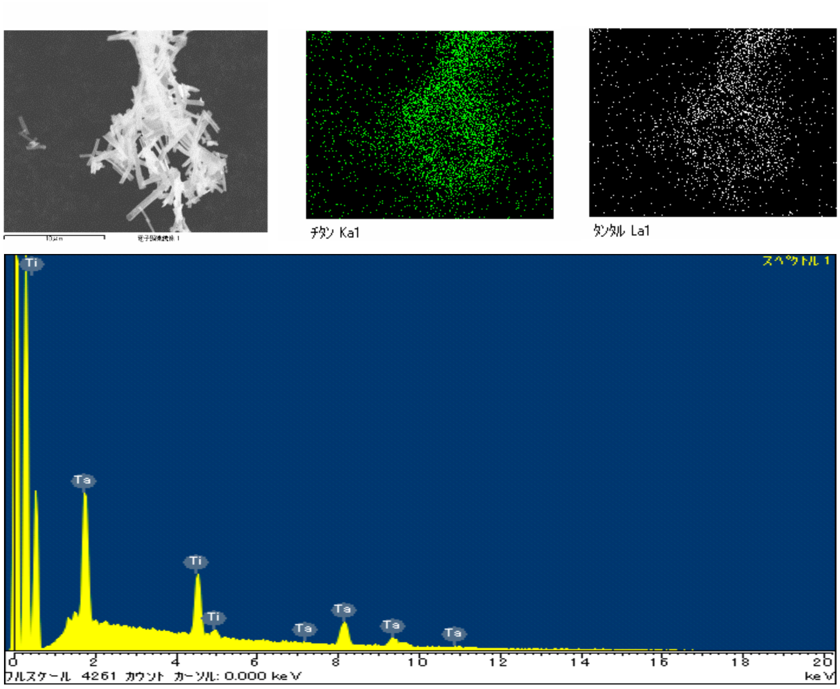

Figure S5. EDX spectra and element mappings of Ta_2O_5/TiO_2 hybrid nanotubes. Upper: Ti:Ta = 7:3; lower: Ti:Ta = 6:4. Scale bars are 10 μm.

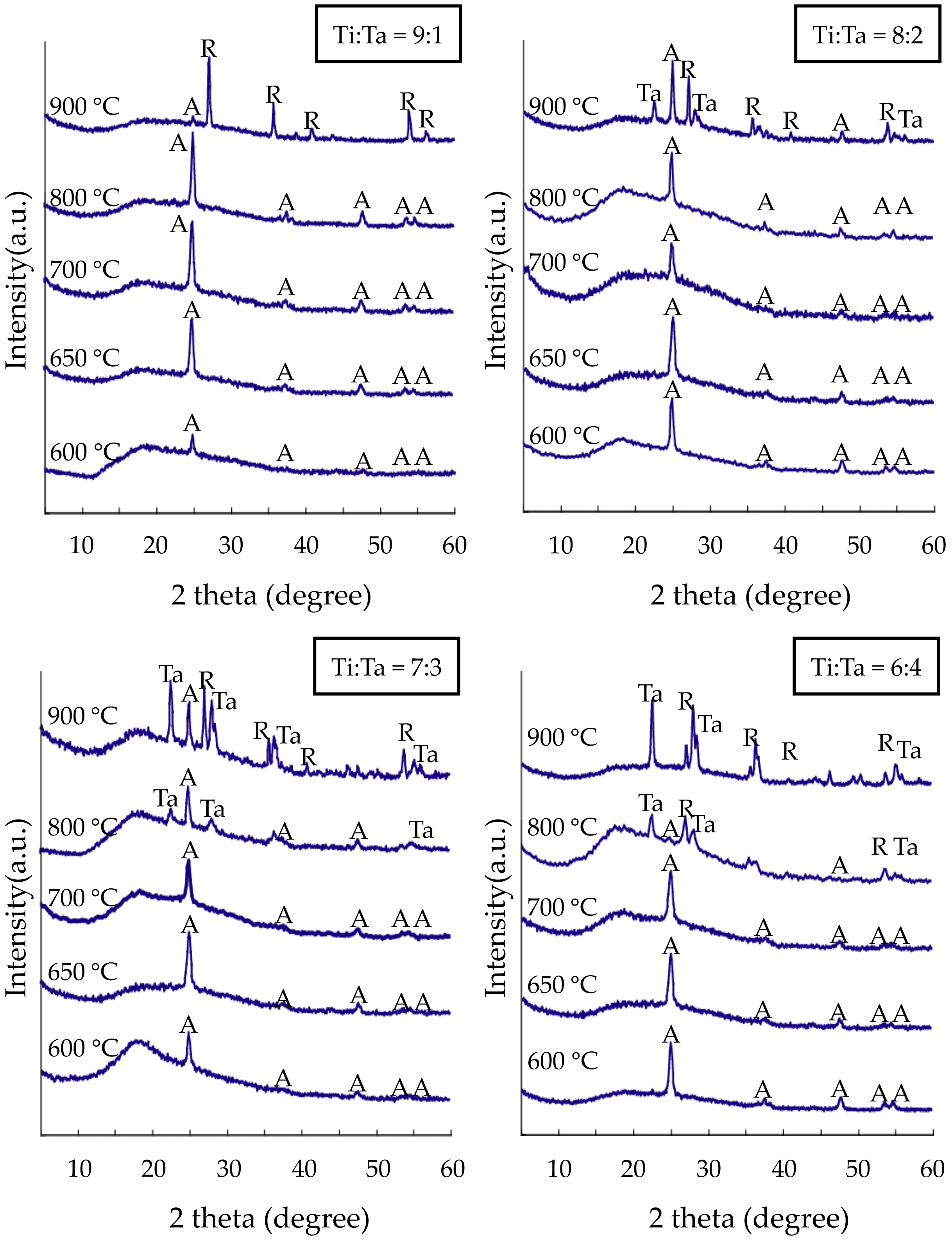

Figure S6. XRD patterns of Ta_2O_5/TiO_2 hybrid nanotubes fabricated at various calcination temperatures.

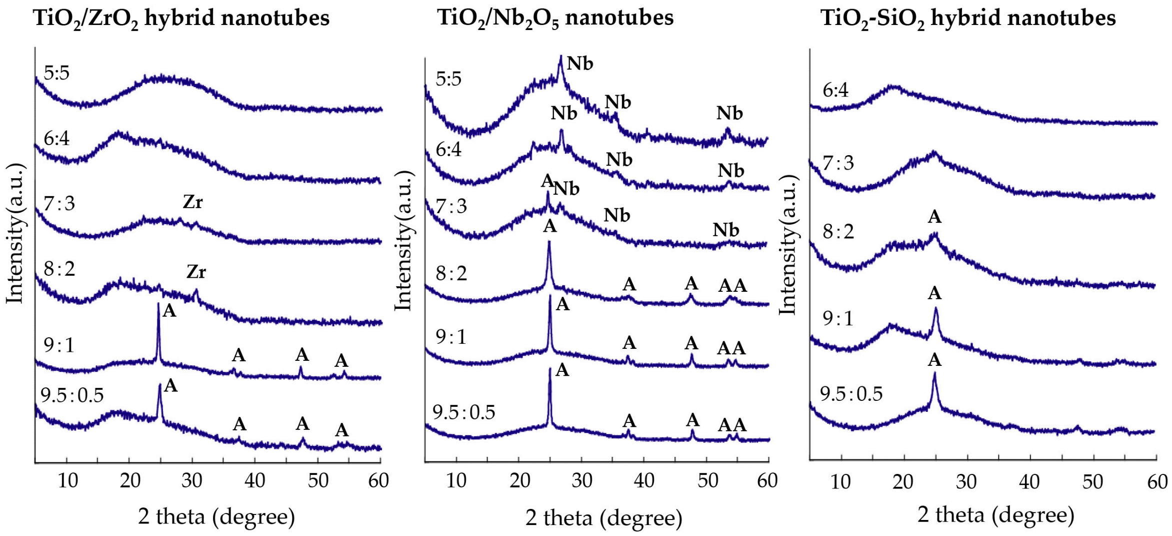

Figure S7. XRD patterns of ZrO2/TiO2, Nb2O5/TiO2, and SiO2/TiO2 hybrid nanotubes.

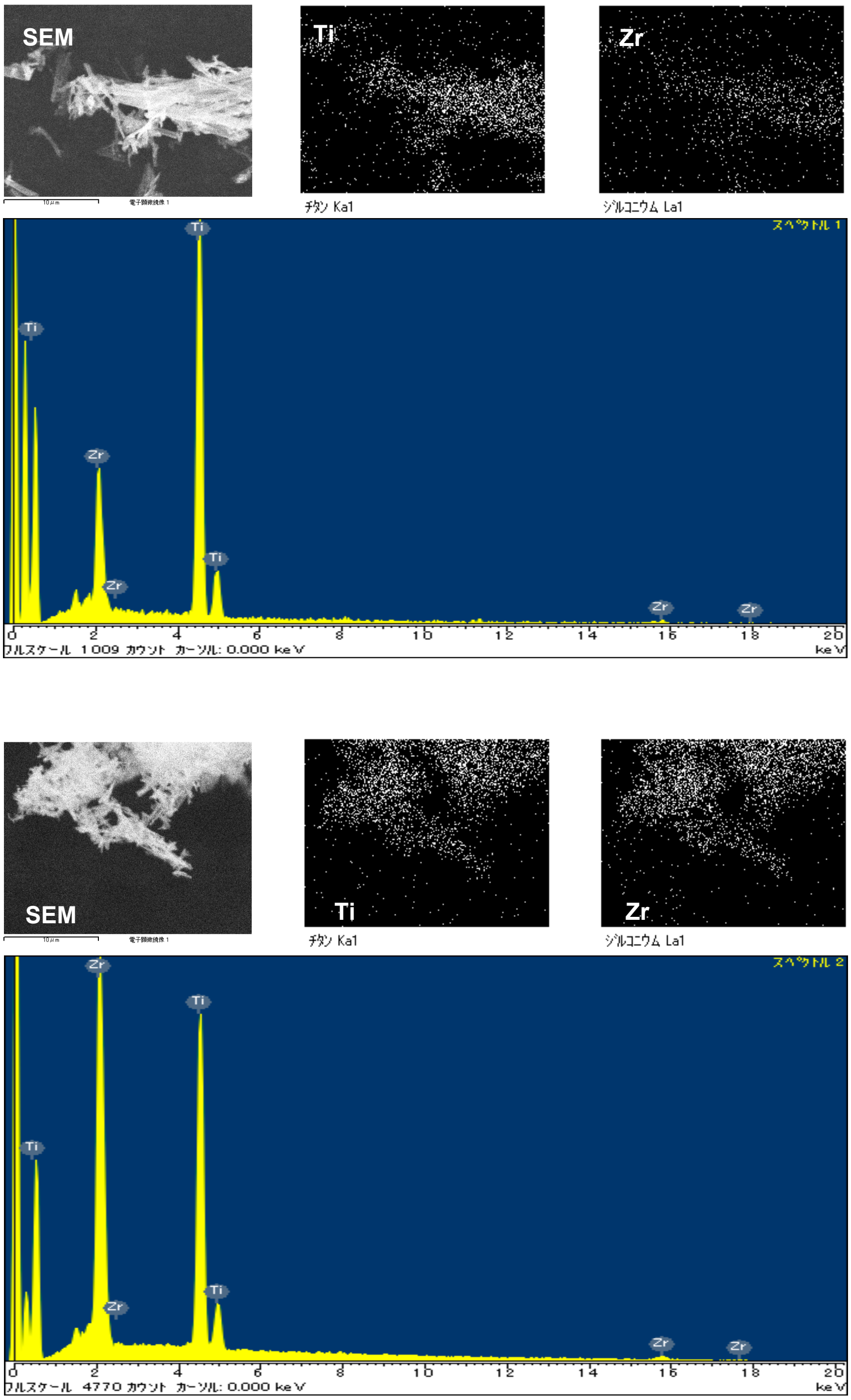

Figure S8. EDX spectra and element mappings of ZrO_2/TiO_2 hybrid nanotubes. Upper: Ti:Zr = 9:1; lower: Ti:Zr = 5:5. Scale bars are 10 μm.

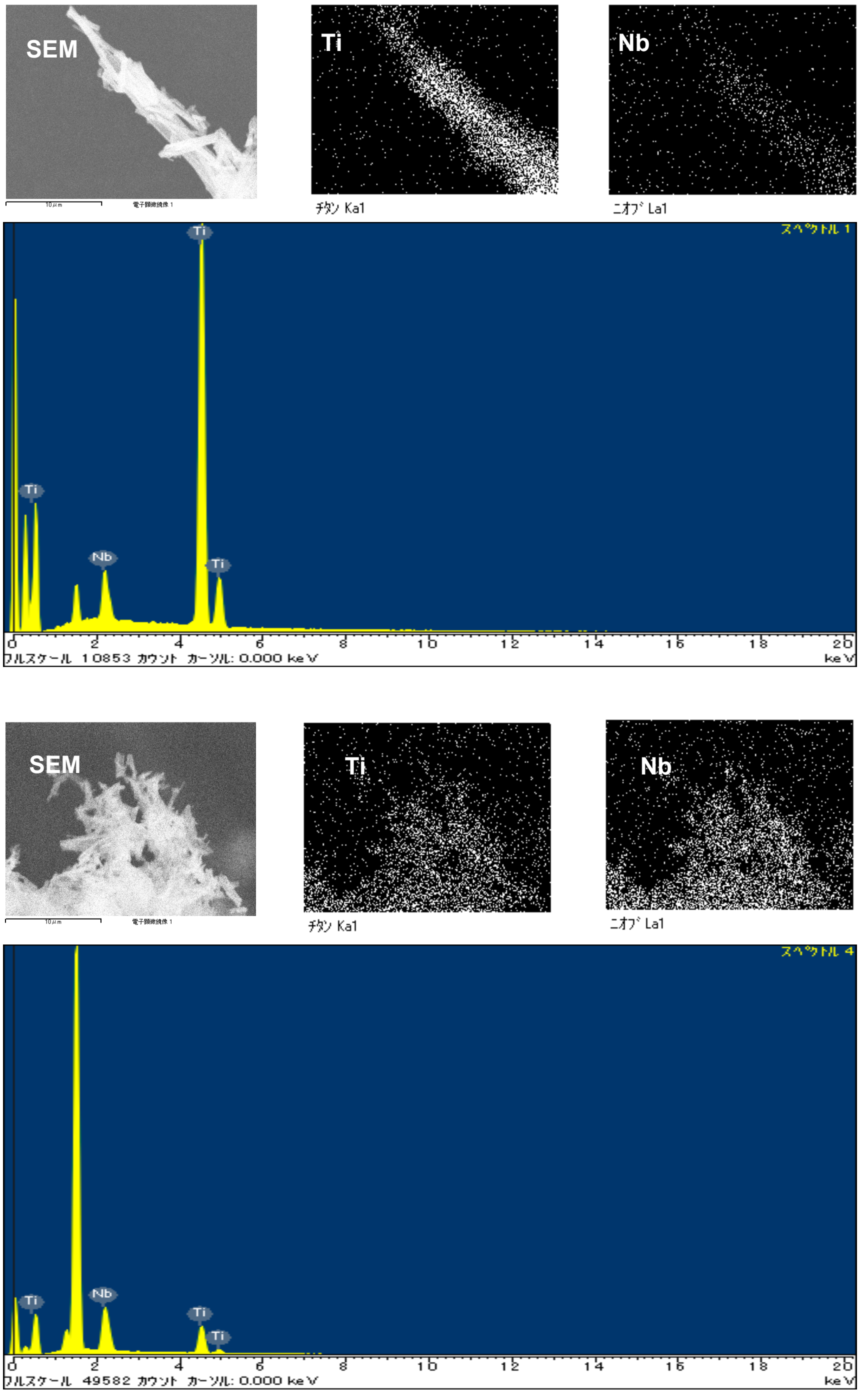

Figure S9. EDX spectra and element mappings of Nb_2O_5/TiO_2 hybrid nanotubes. Upper: Ti:Nb = 9:1; lower: Ti:Nb = 5:5. Scale bars are 10 μm.

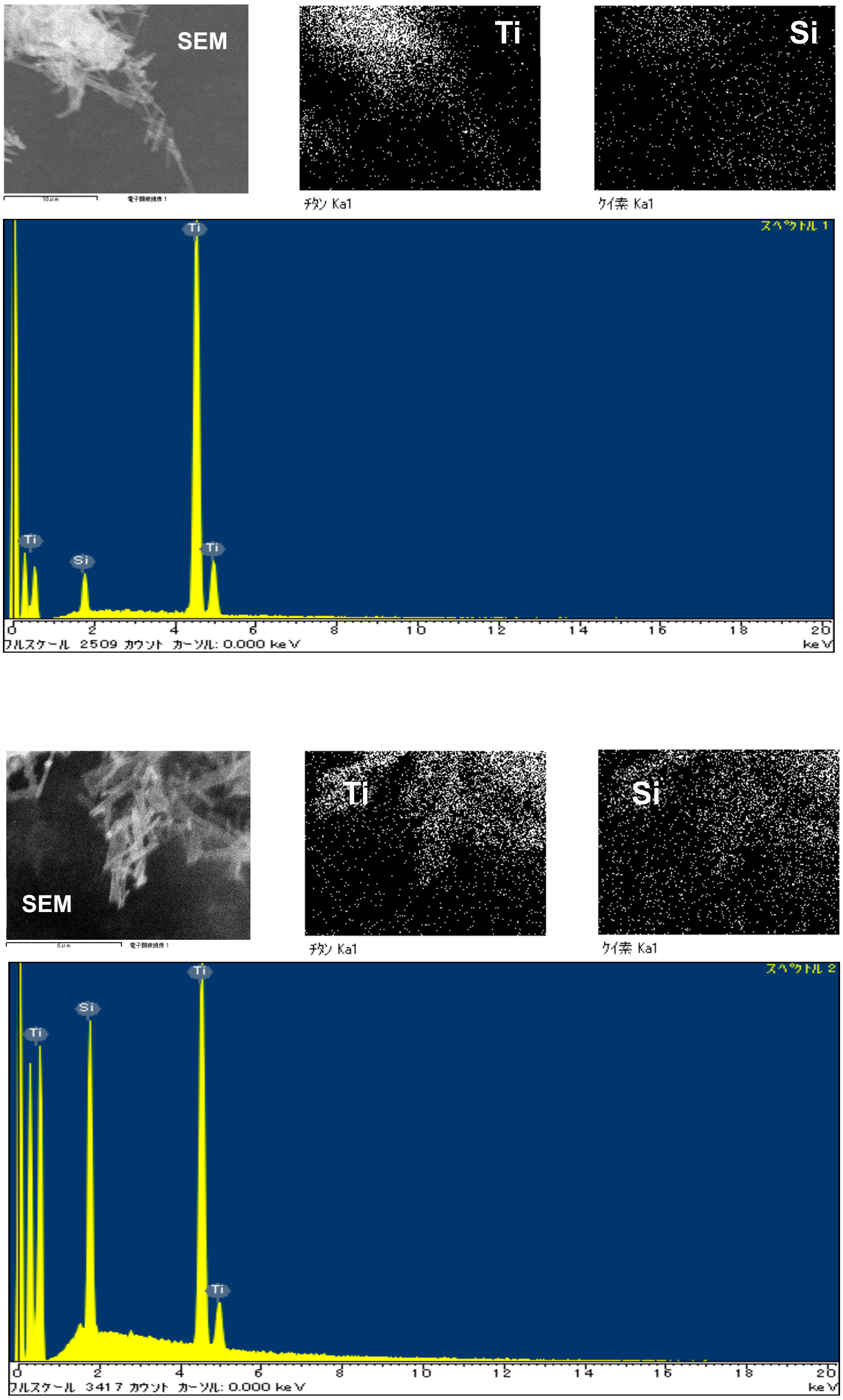

Figure S10. EDX spectra and element mappings of SiO_2/TiO_2 hybrid nanotubes. Upper: Ti:Si = 9:1; lower: Ti:Si = 6:4. Scale bars are 10 μm.

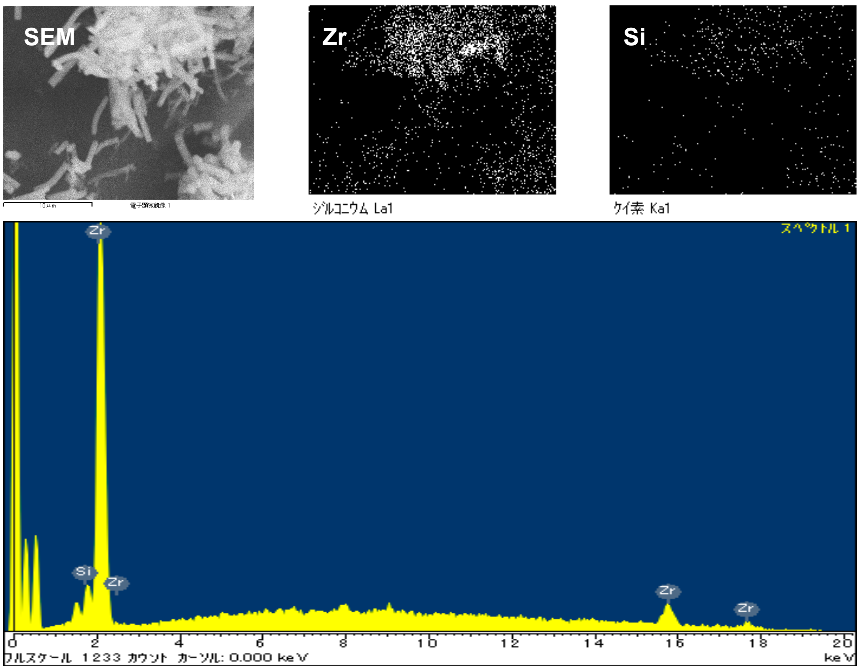

Figure S11. EDX spectra and element mappings of ZrO_2/SiO_2 hybrid nanotube (Zr:Si = 9:1). Scale bars are 10 μm.

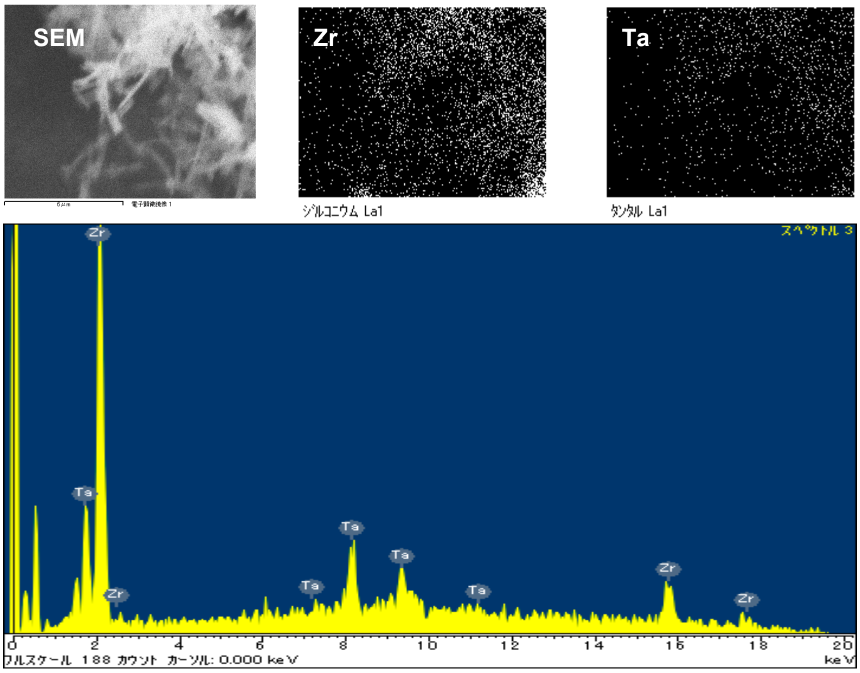

Figure S12. EDX spectra and element mappings of ZrO_2/Ta_2O_5 hybrid nanotube (Zr:Ta = 9:1). Scale bars are 10 μm.
